# Safety Profile and Suicidality Associated with the Use of Esketamine in the Treatment of Major Depressive Disorder in European Countries: An EudraVigilance Database Analysis

**DOI:** 10.3390/ph18050702

**Published:** 2025-05-09

**Authors:** Ilaria Ammendolia, Carmen Mannucci, Emanuela Esposito, Gioacchino Calapai, Mariaconcetta Currò, Paola Midiri, Cristina Mondello, Luigi Cardia, Fabrizio Calapai

**Affiliations:** 1 Department of Chemical, Biological, Pharmaceutical and Environmental Sciences, University of Messina, 98122 Messina, Italy; ilaria.ammendolia@unime.it (I.A.); emanuela.esposito@unime.it (E.E.); 2 Department of Clinical and Experimental Medicine, University of Messina, 98122 Messina, Italy; mariaconcetta.curro@unime.it (M.C.); paola.midiri@gmail.com (P.M.); 3 Department of Biomedical and Dental Sciences and Morphological and Functional Imaging, University of Messina, 98122 Messina, Italy; carmen.mannucci@unime.it (C.M.); cristina.mondello@unime.it (C.M.); fabrizio.calapai@unime.it (F.C.); 4 Department of Human Pathology of Adult and Childhood “Gaetano Barresi”, University of Messina, 98122 Messina, Italy; luigi.cardia@unime.it

**Keywords:** esketamine, pharmacovigilance, adverse reactions, major depressive disorder, treatment-resistant depression, suicide

## Abstract

**Background/Objective:** Major depressive disorder (MDD) is a common mental disorder, with a significant portion of patients developing treatment-resistant depression (TRD). Esketamine is an antagonist of the N-methyl-D-aspartate receptor indicated as a nasal spray in combination with other antidepressants for adults with TRD. Signals of suspected adverse reactions (SARs) to esketamine from the EudraVigilance database in European countries were analyzed for a more defined safety profile of this drug in the real world. **Methods:** SARs to esketamine reported in the data system EudraVigilance were analyzed, and disproportionality analysis for adverse reactions indicating suicidality for esketamine, in comparison to the antidepressants fluoxetine and venlafaxine, was performed. **Results:** Increases in blood pressure (15.4%) and dissociation (15.0%) were the most frequently reported SARs. The sex distribution indicates the prevalence of women, except for increased blood pressure and completed suicide, which were signaled in men, while adults (18–64 years) and elders (65–85 years) were the ages with the largest number of reported adverse reactions to esketamine. The results indicate the existence of a potential increase in the risk of suicide in depressed patients taking esketamine when compared with fluoxetine and venlafaxine. **Conclusions:** Apart from carefulness due to the known limitations of pharmacovigilance research conducted by using data systems of spontaneous signals for SARs, the analysis of data points toward the need for greater attention being paid to the potential risk of suicide following the prescription of esketamine in depressed subjects. In this regard, as regulatory agencies also recommend, patients with a history of suicide-related events or those exhibiting a significant degree of suicidal ideation prior to beginning treatment should receive more careful monitoring during treatment.

## 1. Introduction

Major depressive disorder (MDD) is a common mental disorder. A significant number of patients diagnosed with this disorder do not achieve complete recovery (with the remission phase lasting for at least six months), even in the face of multiple pharmacological trials, developing a form of treatment-resistant depression (TRD) [1]. The concept of depression resistant to antidepressant pharmacological treatment was officially introduced by Lehmann in 1974 [2], and it has been described over time through studies and publications that have outlined its profile [3]. At present, however, there is no precise definition of TRD, which is why clinicians and researchers tend to embrace the postulate formulated at the turn of the century, according to which a subject is resistant to treatment when successive therapies, conducted consecutively with two molecules belonging to different pharmacological classes, do not produce acceptable therapeutic effects even if taken for a sufficient period of time and at an adequate dosage [4]. The concept of TRD is applied in clinical practice when two or more successive treatments with different antidepressant drugs did not work [5].

Pharmacological therapies for TRD have taken two main paths, one with the use of serotonergic drugs and another by prescribing glutamatergic or psychedelic substances such as ketamine and esketamine [6]. It was observed that the racemic mixture (R,S)-ketamine (referred to as ketamine), an NMDA receptor (NMDAR) antagonist, produced antidepressant effects in humans [7,8], with similar behavioral and neurochemical profiles to antidepressant drugs [9], and that NMDA antagonists mimic the effects of antidepressants [10]. Ketamine, derived from phencyclidine, produces a dissociative and anesthetic state in humans that led to its use as an anesthetic and analgesic [11], but its antidepressant effects were not fully realized until decades later [12]. Ketamine induces significant antidepressant effects within a few hours. Due to the rapid antidepressant effects of ketamine compared to the delayed beginning of the impact of traditional antidepressants [13], research on this drug has continued and has revealed its potential drug targets. Mechanism of action seems to be related to the properties of its metabolites. Ketamine is rapidly metabolized, resulting in neuroactive products that likely contribute to its therapeutic effects. It undergoes its first metabolic transformation into (R,S)-norketamine in the liver. Subsequently, (R,S)-norketamine can be converted to (R,S)-dehydronorketamine or (R,S)-hydroxynorketamine [14]. Esketamine, the S-enantiomer of racemic ketamine, is a nonselective, noncompetitive antagonist of NMDAR [15,16]. Through NMDAR antagonism, esketamine produces a transient increase in glutamate release, resulting in increased α-amino-3-hydroxy-5-methyl-4-isoxazolepropionic acid receptor stimulation and subsequent increases in neurotrophic signaling. Thus, it seems to contribute to the restoration of synaptic function in brain regions involved in the regulation of mood and emotional behavior [17]. The restoration of dopaminergic neurotransmission, involved in reward and motivation and the reduced stimulation of brain regions involved in anhedonia, may contribute to the rapid response [18]. In 2019, the European Medicines Agency (EMA) approved esketamine as a nasal spray that delivers a total of 28 mg of esketamine in two actions (one in each nostril), in combination with a selective serotonin reuptake inhibitor (SSRI) or a serotonin and norepinephrine reuptake inhibitor (SNRI), for adults with treatment-resistant major depressive disorder (MDD) who have not responded to at least two different treatments with antidepressants in a current moderate to severe depressive episode. Furtherly, co-administered with oral antidepressant therapy, it is indicated in adults with a moderate to severe episode of MDD, as an acute short-term treatment, for a rapid reduction in depressive symptoms, which, according to clinical judgement, constitute a psychiatric emergency. The most commonly observed adverse reactions in patients treated with esketamine based on data collected for clinical studies used for the market registration of the drug were dizziness (31%), dissociation (27%), nausea (27%), headache (23%), somnolence (18%), dysgeusia (18%), vertigo (16%), hypoesthesia (11%), vomiting (11%), and increased blood pressure (10%) [19].

Monitoring the safety of drugs after their approval and consequent release in the market is a key point in defining a positive benefit–risk ratio in the life cycle of a drug. This surveillance is performed through pharmacovigilance, an activity defined as “the science and activities relating to the detection, assessment, understanding and prevention of adverse effects or any other drug-related problem” [20]. The aims of pharmacovigilance are the prevention of injury from adverse reactions in humans caused by the use of drugs after their approval, the promotion of safe and effective use of drugs, and the provision of ready information about the safety of medicinal products to the public, patients, and healthcare professionals [21].

The aim of the study is to obtain an updated definition of the post-marketing safety profile related to the use of the drug esketamine in subjects affected by MDD through the analysis of spontaneous reports of suspected adverse reactions identified in the European database EudraVigilance. Another aim of this study is to analyze, through a disproportionality analysis, calculated by using the reporting odds ratio, the comparison of adverse reactions related to suicidality in patients who were prescribed the drug esketamine with those who were prescribed fluoxetine [22] or venlafaxine [23], which are drugs associated with the prescription of esketamine, as indicated in its market authorization [19].

## 2. Results

**Figure 1:**
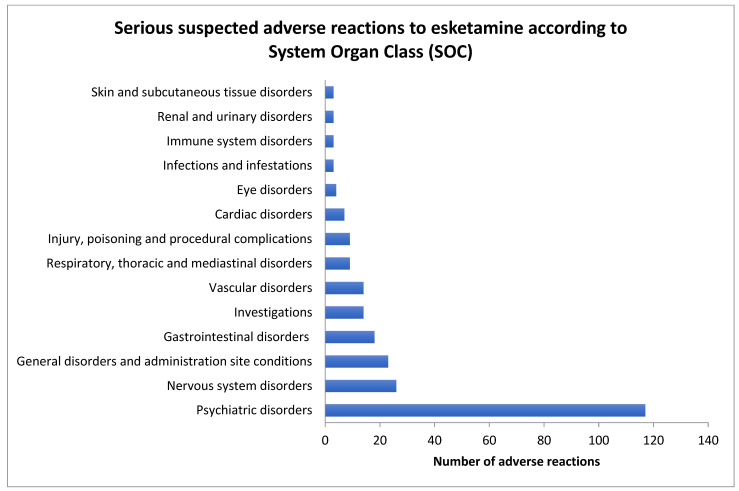
Serious suspected adverse reactions to esketamine signaled in the European Economic Area and the United Kingdom in the years 2019–2024 according to the System Organ Class (SOC) levels. Total number of cases is 265.

A total of 751 ICSRs related to esketamine were identified in the Eudravigilance database as signals of adverse reactions to esketamine in the period from 2019 (the year of market entry of the drug) to 31 December 2024. Of these reports, 265 were categorized as serious cases (35.3% of the total number of ICSRs) and 486 as non-serious cases. The distribution by sex shows that of the 486 non-serious cases, 306 reports were related to female subjects (62.4%) and 168 to male subjects (sex was not indicated in the remaining cases). Similar percentages were observed with serious cases, comprising 169 female cases (63.8%) and 96 male cases, thus indicating that both serious and non-serious cases are more frequent in women. However, among serious cases, death is a result in 27 cases, of which 18 are men (66.7%). Most of the cases of death were caused by suicide. A total of 17 total cases of death were caused by suicide (63% of cases of death); 12 were men and 5 women, showing a larger percentage of suicide in men (70.6%). The frequency in descending order of single adverse reactions indicates that increased blood pressure, dissociation/dissociative disorder, anxiety and completed suicide are more often related to esketamine than other serious events (Figure 1).

Data are presented as the number of single adverse reactions. Only groups of adverse reactions signaled more than two times are considered.

The statistical analysis of sex distribution shows that the adverse reactions of increased blood pressure and completed suicide are signaled more frequently for men. Dizziness is an adverse reaction to esketamine involving prevalently women. There is no statistical difference in the sex distribution of the other adverse reactions to esketamine (Table 1).

The age distribution of cases shows that adults (18–64 years) and the elderly (65–85 years) are more affected. This is a predictable result, because at these ages, esketamine is more naturally prescribed for depression. No statistical difference is revealed by data analysis, except for increased blood pressure being more signaled in the elderly (65–85 years). Moreover the adverse reaction “hallucination” is only reported in adults (18–64 years) (Table 2).

Cases of death not including suicide, among all serious SARs caused by esketamine, are 3.8%, with prevalence in men (2.3%) in comparison to women (1.5%). Most of these fatal events were observed in the group of adult patients (Table 3).

Diagnoses related to deaths associated with esketamine comprised three cases of sudden death and other single diagnoses such as respiratory failure, pulmonary embolism, and myocardial infarction. The results regarding groups of adverse reactions aggregated according to SOC levels show that signals for adverse reactions to esketamine produced an increased ROR in comparison to signals for adverse reactions to both fluoxetine and venlafaxine. In particular, these parameters are increased for psychiatric disorders, vascular disorders, and immune system disorders among the adverse reactions to esketamine (Table 4).

Most psychiatric disorders potentially caused by esketamine are represented by single adverse reactions such as dissociation/dissociative disorders, followed by “suicidal ideation, anxiety, and completed suicide. The vascular disorders signaled for esketamine are for the most part dizziness and hypertension, while for immune system disorders, several cases of anaphylactic reactions were identified (Table 1 and Table 2). The ROR of cases signaling the single serious adverse reactions of suicidal ideation, suicide attempts and complete suicide, suspected to be caused by esketamine in comparison with the two antidepressant drugs fluoxetine and venlafaxine, shows an increased potential risk of suicidal ideation with esketamine compared to fluoxetine (ROR 2.94; C.I.1.75–4.94) and a more increase of potential risk in comparison to venlafaxine (ROR 5.25; C.I. 3.15–8.73). A light increase in potential risk was also detected in suicide attempts with esketamine in comparison to venlafaxine (ROR 1.05; C.I. 0.59–1.86). A significant increase in the potential risk with esketamine was detected with the calculation of signals regarding completed suicide, comparing signals with those of fluoxetine (ROR 8.05; C.I. 3.55–18.3) and even more with venlafaxine (ROR 10.58; C.I. 5.08–22.04). The comparison of signals for suicidal ideation, suicide attempts, and completed suicide between fluoxetine and venlafaxine showed only a moderate increase in potential risk with fluoxetine treatment (Table 5).

## 3. Discussion

The approval of N-methyl-D-aspartate/glutamate receptor (NMDAR) antagonists by the FDA and EMA for therapy for depression has opened a large debate in the fields of psychiatry and psychopharmacology. Their use is based on the observation of depression-related alterations in glutamate synaptic signaling, such as the reduced amplitude of sensory-evoked potentials and the reduction in cortical functional connectivity [24], reduced synaptic density, and disrupted synaptic glutamate homeostasis [25,26], together with the elevation of extracellular glutamate levels, overstimulating extrasynaptic N-methyl-D-aspartate (NMDA) receptors (NMDAR). In light of these findings, it is believed that, if used appropriately, the efficacy of NMDAR antagonists, including esketamine, can have a positive impact on the lives of people suffering from depression and, as a consequence, on the public health burden associated with TRD [27].

MDD is highly prevalent and is associated with a substantial burden and the high economic costs represented by drugs, diagnostic exams and laboratory investigations, and absence or poor performance at work [28]. The largest portion of subjects affected by MDD are not sufficiently responsive to first-line treatments, and in a significant part of them, the failure of multiple antidepressant pharmacological treatments is detected, resulting in what is called TRD [29]. After its release into the drug market, many studies investigated the clinical effects of esketamine. Esketamine nasal spray has been studied in the long-term trials SUSTAIN-1 and SUSTAIN-3 conducted on patients with TRD. A subgroup analysis of SUSTAIN-3 evaluated 96 patients with TRD who received a second induction and maintenance treatment with esketamine plus an oral antidepressant after a relapse in SUSTAIN-1. The authors observed that patients with TRD benefitted from receiving a second induction and maintenance treatment with ESK, and no new safety signals were identified [30].

Despite being approved by the FDA and EMA, the utility of esketamine nasal spray as an add-on treatment with an antidepressant in MDD is still debated. A meta-analysis was conducted on nine randomized clinical trials comparing esketamine nasal spray versus control in MDD or TRD, with the aim of evaluating if this treatment could rapidly, effectively, and persistently control depressive symptoms in short- and long-term periods. The authors concluded that esketamine nasal spray in conjunction with an antidepressant effectively controls short-term and long-term depressive symptoms in MDD and RTD, supporting its clinical utility [31].

Finally, the most recent study investigating the efficacy of esketamine nasal spray for TRD is a review and meta-analysis including five randomized clinical trials in which RCTs are included in the meta-analysis. In this study, the authors observed that adverse events, such as dizziness and nausea, were more common following esketamine treatment, but they were generally well tolerated by patients, and the authors concluded that esketamine nasal spray is beneficial to improve the efficacy of treatment-resistant depression [32].

Moreover, the post-marketing occurrence of respiratory depression associated with esketamine treatment has been signaled, but no adverse events of respiratory depression were reported in ESK phase 3 clinical trials. Moreover, in a study involving fifty cases of depressed patients treated with esketamine matching the definition for respiratory depression, only eight of these showed a stronger association with esketamine therapy [33]. Another study based on a total of 2907 female reports and 1634 male reports on esketamine extracted from the databank Food and Drug Administration on Adverse Event Reporting System (FAERS) showed that completed suicide, decreased therapeutic product effects, urinary retention, and hypertension were common in men [34]. The analysis of data on neurological adverse events caused by esketamine and extracted from FAERS suggests that a higher dose of esketamine, antidepressant polypharmacy, and combination treatment with benzodiazepines or somatic medications are more likely to be risk factors related to adverse events severity, whereas age and sex are not [35].

Our post-marketing analysis of SARs contained in the ICSRs sent to EudraVigilance only partially confirms the data used for authorization reported in the official summary of product characteristics. In the same document (associated with the market release of the drug), potential adverse reactions are listed according to the designated system organ classes (SOC). This classification reported as very common adverse reactions (≥1/10) the adverse reaction of dizziness and the psychiatric disorder of dissociation, and in the category of nervous system disorders, it included symptoms such as headache, somnolence, dysgeusia, and hypoesthesia. Other adverse reactions reported as very common were nausea, vomiting, and increased blood pressure. Our post-marketing analysis modifies this view and, as a consequence, shows a different safety profile of esketamine.

In the EudraVigilance data system used for this study, psychiatric disorders, nervous system disorders, vascular disorders, and investigations (including increased blood pressure) are the groups of adverse reactions most frequently signaled for esketamine. Among the single adverse reactions, increased blood pressure (signaled in 16.2% of all the ICSRs) and dissociation/dissociative disorders (signaled in 15.8% of all the ICSRs) are those more frequently reported. Sex distribution indicates that women are prevalently implicated in ICSRs except for increased blood pressure and completed suicide, which are more signaled for men, while age distribution shows that adults (18–64 years) and elders (65–85 years) are the ages with the largest number of reported adverse reactions to esketamine. Patients with TRD treated with esketamine nasal spray commonly experience transient symptoms of dissociation. Dissociative disorders, such as feelings of detachment from the environment, also cause considerable anxiety for patients [36]. Adverse reactions signaled as anxiety following esketamine prescription are also repeatedly reported in EudraVigilance. The increase in blood pressure is confirmed by another study carried out by analyzing the post-marketing safety signals of esketamine nasal spray, downloading data from the US FAERS from Q1 2019 to Q2 2023, and using methods of disproportionality. The most frequently observed adverse events were dissociation, sedation, and hypertension [37].

Even though adverse reactions are frequently signaled in relationship to esketamine prescription, increased blood pressure is generally considered transient, asymptomatic, and not associated with serious cardiovascular safety sequalae [38]. In addition, cardiovascular effects characterized by increased blood pressure are partially expected based on the esketamine sympathomimetic effect [18].

Previously, a randomized placebo-controlled trial investigated the efficacy and safety of 28, 56, or 84 mg of esketamine intranasal spray twice weekly for 4 weeks in 138 adults ≥ 65 years old with depression. The trial investigators reported a transient elevation in mean blood pressure in participants receiving esketamine that peaked at 40 min post-treatment and resolved in 2 h in about 80% of participants [39]. Our data also reveal that the potential risk of increased blood pressure can be significantly augmented in the elderly.

More recently, an increase in reporting of hepatobiliary adverse effects associated with esketamine use supports the recommendation for the periodic monitoring of hepatic function through laboratory testing and clinical surveillance. Safety information purchased by the FDA supports the general leading advice to periodically monitor liver function in patients chronically receiving esketamine [40]. The association between esketamine and eye disorders such as cataracts and glaucoma has been studied by collecting data from FAERS. RORs of 1.31 with 95% C.I. 0.63–2.72 for cataracts and 2.19 with 95% C.I. 0.12–39.76 for glaucoma were found as related to esketamine adverse reactions, thus suggesting a greater potential risk for eye disorders [40]. Both liver and eye disorders related to esketamine use have been observed by other investigators, and we have also recorded how these disorders are related to the prescription of this drug. They deserve to be discussed and studied in depth, but this is beyond the scope of this article.

Given that TRD is associated with chronic depression, suicidal behaviors, and a reduction in quality of life [41] and given that the rate of suicidality, including completed suicide, is disproportionately higher in TRD populations [42], the analysis of data founded in EudraVigilance about the adverse reactions suicidal ideation, suicide attempts, and completed suicide in association with esketamine prescription deserves a further and separate discussion. The scientific literature on this argument reports several studies facing the problem of suicidality linked to esketamine use. Multiple severe adverse events during the long-term treatment of 1 year with esketamine, including anxiety, delusional content, delirium, and suicidal ideation, have been reported. These adverse events were found in 0.06% of the sample, including a total of three deaths due to suicide. However, according to the authors of the report, only on the basis of these results, the attribution of suicide to esketamine treatment is difficult due to insufficient consistency [43]. In antithesis to these results, recent evidence indicates that esketamine reduces measures of suicidality in people with TRD, even though, in this case, the same authors declared that it is not clear if individuals experience the worsening of preexisting suicidality with this drug. By using data from the Food and Drug Administration Adverse Event Reporting System (FAERS) database, they collected data from 2019 to 2023 for reports of suicidal ideation, suicidal depression, suicidal behavior, suicidal attempts, and completed suicide in association with esketamine. The results showed a higher ROR for suicidal ideation (ROR 7.58, 95% CI 6.34–9.07) and suicidal depression (ROR 14.19, 95% CI 1.80–112.07), but a lower ROR for suicide attempts (ROR 0.57, 95% CI 0.48–0.67) with esketamine. The limitations of the FAERS database prevented any determination of a causal effect between new-onset suicidality to esketamine. Consequently, the authors concluded that the ROR for suicide attempts with esketamine could not be interpreted as a direct therapeutic effect [44].

A previous study, also using the FAERS database and analyzing data on esketamine-related adverse events from March 2019 to March 2020, estimated the ROR and information component for esketamine-related adverse events at ≥ 4 counts. After comparing data with those of the antidepressant venlafaxine, safety signals for esketamine were detected for self-injurious ideation and suicidal ideation, but not for completed suicide, and for suicide attempts. The authors of the study concluded that esketamine may carry a clear potential for serious adverse events, which requires urgent clarification [45].

More recently, two cases of the deterioration of depressive symptoms and suicidal ideation were reported in patients treated with esketamine for TRD. The two cases initially responded well to intranasal esketamine but later deteriorated rapidly, with a worsening of depressive symptoms and suicidal ideation. Upon discontinuing esketamine, both patients clinically improved and showed a reduction in suicidal ideation [46]. As suggested by the investigators publishing these cases, even if the identification of paradoxical reactions is difficult in TRD patients with highly resistant responses to treatment and suicidal ideation, it could be relevant to examine the prevalence of this phenomenon with the aim of ameliorating the knowledge of the real complications potentially associated with treatment with esketamine.

In a randomized, double-blind placebo-controlled clinical trial, the effects of subclinical doses of esketamine on postpartum depression and pain following elective cesarean sections have been investigated. Based on the results, the authors concluded that esketamine did not reduce the incidence of postpartum depression at 14 days postpartum but did significantly lower pain scores on the visual analog scale at 24 h post-surgery. Moreover, a group of women treated experienced temporary increases in adverse reactions and sedation shortly after administration [47].

Furthermore, a systematic review and meta-analysis of the available data on esketamine’s efficacy against depression and suicidality and on its undesired effects have been conducted through the analysis of 87 articles. The results showed that at weeks 2–4, randomized controlled trials were mostly negative or failed, but a weak significant positive effect for depression was detected at weeks 2–4. However, the effect size concerning suicidality was not significant at any time point. In light of these results, the authors concluded that esketamine’s efficacy as an add-on to antidepressants is modest in TRD patients and is absent against suicidality [48]. These aspects need to be considered in light of esketamine’s abuse potential and the fact that long-term effects are still not fully known.

Analysis of sex distribution indicates the adverse reaction of completed suicide is signaled more frequently in men. Considering the differences in the pharmacokinetics and hormonal status in patients of different genders, the difference in sex distribution observed in our analysis is not surprising. In this regard, a recent study, carried out on data from FAERS on signals of adverse reactions to esketamine of 2907 females and 1634 males, reported ROR results showing that completed suicide was more common in men. Additionally, 552 other female and 653 other male ketamine reports were analyzed with an ROR revealing that women were more likely to develop suicidal ideation [34].

Data obtained from other authors through the database EudraVigilance showed that cases of mania and hypomania were reported disproportionately more frequently with intranasal esketamine in comparison to all the rest of the drugs in the database [49]. Another recent analysis of data from the database EudraVigilance, performed with the aim of assessing psychiatric adverse reactions, including suicide-related events, associated with the prescription of commonly used antidepressants indicated that venlafaxine was associated with the highest number of suicides, followed by citalopram and fluoxetine [50]. Since esketamine is used in combination with an SSRI or SNRI drugs, a comparison of data about suicidality in the present work was performed against the antidepressants fluoxetine and venlafaxine. The analysis, even though strongly influenced by the different times of market availability between esketamine and the two drugs fluoxetine and venlafaxine, which have been available for several decades, while esketamine was authorized by the EMA in 2019 [51], shows a significant increase in the potential risk of suicidality for this last substance. ROR is a disproportionality measure that can be used to confirm the association between a drug and a certain adverse event. In our analysis, the association between suicidality and esketamine seems to be more pronounced than the same association with the drugs fluoxetine and venlafaxine, with the probability for association with completed suicide increasing about four times and five times in comparison to fluoxetine and venlafaxine, respectively. Furthermore, as mentioned above, the rate of suicidality is already high in TRD patients, and this makes it more difficult to assess how much esketamine might affect a frequency that is already elevated. Moreover, esketamine is a drug with a low affinity for NMDAR, and it can also bind to other targets, such as opioid receptors. For this reason, the precise mechanism of action for its antidepressant properties remains debated, as does its potential for misuse [52]. It is believed that esketamine’s psychoactive effects have a role either in the antidepressant effect or in its potential for abuse. Data from the pharmacovigilance approach have suggested that they are a bad experience for certain individual patients, while they are desired by others [53]. Finally, an indication of the potential risk of suicidality is emerging, even if the drug esketamine is prescribed for individuals affected by depression and naturally exposed to suicide and it cannot be excluded that esketamine is indicated for TRD, a condition even more at risk for suicidality, since, compared with other patients with MDD, those with TRD have been shown to have carried out more suicide attempts [54]. Moreover, according to other authors, it is very difficult to differentiate between suicidal ideation as part of depression intended as a disease with respect to suicidal ideation potentially related to lack of esketamine effectiveness or as a reaction induced by esketamine [55].

Anyway, even though we conducted this research keeping well in mind the ethical principles on which pharmacovigilance is based, primarily aimed at safeguarding the well-being of patients and promoting public health, the results of this study need to be interpreted with care due to the known limitations of pharmacovigilance research conducted by using data systems of spontaneous signals for drugs adverse reactions. These limitations include the lack of a denominator, under-reporting, the minor quality of information, and the uncertainty of causal relationships, and, finally, due to the lack of information in the EudraVigilance database, it is difficult to control confounding factors such as comorbidities or, sometimes, dosage and frequency duration of exposure, which may have an influence on health. Furthermore, although care and attention were taken to identify and remove duplicates, they may still exist in the data.

## 4. Materials and Methods

EudraVigilance is a database containing suspected adverse reactions (SARs) related to medicines authorized for the market or currently undergoing clinical trials in the European Union (EU). In this data system, SARs are traceable in individual cases (Individual Cases Safety Reports; ICSRs) signaled by national drug regulatory authorities in the EU or by marketing authorization holders. It is noteworthy that EudraVigilance collects reports of “suspected” adverse reactions, meaning unwanted medical events that have been observed following the use of a medicine, but which are not necessarily related to or caused by the medicine itself [56].

### 4.1. Design of the Study

In this study, ICSRs reporting SARs that occurred in patients to whom esketamine was prescribed, signaled from 1 January 2019 to 31 December 2024, were collected and analyzed. The public version of the EudraVigilance database was used, and the collection of data on SARs was conducted according to the following inclusion criteria: only serious SARs and reports sent only from healthcare professionals in cases regarding all ages (from 0 to >85 years) and signaled from the European Economic Area, including the UK. Reports submitted in EudraVigilance originated from EEA and non-EEA countries, but for this study, we selected only reports originating from the European Economic Area (EEA). We mention the UK separately because in EudraVigilance, it continues to be included within the EEA. Signals were excluded from the analysis when categorized as not serious, reported by not healthcare professionals, or coming from non-European countries. For all cases, information was provided on patient characteristics (age group and sex), the type of adverse reaction (often more than one for each ICSR), and the qualification of the primary source. The terms “sex” and “gender” are used interchangeably here because only the field containing the term “sex” is available in EudraVigilance; consequently, the information collected refers to biological sex [57]. Regarding the criteria for the collection of data from ICSRs, SAR selection was based on the Medical Dictionary for Regulatory Activities (MedDRA). MedDRA is an international standardized and clinically validated medical terminology used by regulatory authorities and the biopharmaceutical industry. It is used to code cases of adverse effects in pharmacovigilance databases and to facilitate searches in databases on adverse drug reactions. For the present study, each ICSR was analyzed, and every mentioned SAR was extracted and counted from every single case. Single serious adverse reactions were described using the so-called “Preferred terms” (PTs) listed in MedDRA. A PT is a distinct descriptor (single medical concept) for an adverse symptom or sign. We selected all the PTs that were recorded in the ICSR, and we counted them all and analyzed the frequencies for each one. Two or more PTs with overlapping clinical meanings were aggregated to avoid unnecessary duplicate PTs with the same connotation. MedDRA has a hierarchy of terms to describe adverse reactions. Adverse reactions were also grouped under the terms of the SOC (System Organ Classification) level in the MedDRA hierarchy such as musculoskeletal and connective tissue disorders and vascular disorders. Each single PT has been associated with the corresponding SOC level by using the MedDRA terminology reported by the National Center for Biomedical Ontology (NCBO). The SOC system organ classification is the highest level of the hierarchy that captures the broadest concept useful for retrieving data. It is a way of grouping medical terms based on body systems or functions [58].

### 4.2. Data Analysis

The source of data extraction is a line listing a structured table, where each row represents an ICSR and each column represents a specific data point associated with that case. The data were analyzed by aggregating the PTs of individual reports to a higher level of the MedDRA hierarchy by merging individual serious SARs in the SOC level (e.g., nausea and vomiting are classified in the same group as Gastrointestinal Symptoms). Only reports classified as serious were analyzed. In accordance with the E2D guidelines of the International Council for Harmonization, ICSRs are classified as serious if they are life-threatening, have resulted in death, have resulted in/prolonged hospitalization or disability, or are related to a congenital anomaly/birth defect or other medically important condition. The adequate stratification of signals by sex groups was performed to avoid biases caused by confounding effects and to analyze these two variables separately. Age and sex distribution were analyzed as ratio of relative risk. Duplicate and incomplete ICSRs were excluded from the analysis. A duplicate search was conducted based on the detection in the dataset of similarities in the adverse reaction, age, sex, suspected/interacting medicinal products, and EudraVigilance local report number. A disproportionate analysis of the potential association of suicidality with esketamine, fluoxetine, and venlafaxine was performed by using the following list of PTs: suicidal ideation, suicide attempts, and completed suicide.

### 4.3. Statistics

A descriptive statistical analysis and a disproportionality analysis based on the reporting odds ratio (ROR) were performed. The ROR is used here to establish the strength of disproportionality. An ROR equal to 1 indicates the absence of a signal; conversely, an ROR greater than 1 indicates a signal and the existence of an association. The higher the ROR, the stronger the association. The ROR is statistically significant when the lower limit of its 95% CI is greater than 1 [59,60]. All statistical analyses were completed using SPSS statistical software, version 29.0 (SPSS, IBM, Armonk, NY, USA).

## 5. Conclusions

An analysis of data on adverse reactions to esketamine in treatment-resistant depression present n EudraVigilance points towards the need for greater attention in the prescription of esketamine in depressed subjects. In particular, the significant increase in the probability of suicidality risk that could arise from the use of this drug is of particular concern. In this regard, as regulatory agencies recommend, patients with a history of suicide-related events or those exhibiting a significant degree of suicidal ideation prior to beginning treatment should receive more careful monitoring during treatment.

## Figures and Tables

**Table 1:** Sex distribution of serious suspected adverse reactions to esketamine signaled in the European Economic Area and the United Kingdom in the years 2019–2024. The total number of Individual Cases Safety Reports (ICSRs) is 265.

Adverse Reaction	Male Cases Number and %	Female Cases Number and %	Male and Female Cases	% of All ICSRs	Significance Level (P)
Increased blood pressure	23 (53.5%)	20 (46.5%)	43	16.2%	0.016410 *
Dissociation/dissociative disorder	14 (33.3%)	28 (66.6%)	42	15.8%	0.802393
Suicidal ideation	9 (34.6%)	17 (65.4%)	26	9.8%	0.972193
Anxiety	5 (21.7%)	18 (78.3%)	23	8.7%	0.198565
Dizziness	2 (11.1%)	16 (88.9%)	18	6.8%	0.041125 *
Completed suicide	12 (70.6%)	5 (29.4%)	17	6.4%	0.005334 *
Drug ineffective	5 (29.4%)	12 (70.6%)	17	6.4%	0.685521
Suicide attempt	3 (21.4%)	11 (78.6%)	14	5.0%	0.369206
Loss of consciousness	6 (46.1%)	7 (53.8%)	13	4.9%	0.639931
Hallucination	4 (33.3%)	8 (66.7%)	12	4.5%	0.925157
Generalized tonic–clonic seizure	4 (50.0%)	4 (50.0%)	8	3.0%	0.653024
Diplopia	2 (28.6%)	5 (71.4%)	7	2.6%	0.977208
Aggression	0 (0%)	5 (100%)	5	1.9%	N.A.
Bradycardia	4 (80.0%)	1 (20%)	5	1.9%	0.112688

Data are presented as number of cases and percentage of single adverse reactions. Only adverse reactions signaled more than 5 times are included in the table. N.A. = not applicable; * = *p* < 0.05 vs. male or female cases.

**Table 2:** Age distribution of individual case safety reports (ICSRs) signaling more frequent serious suspected adverse reactions (SARs) associated with esketamine use in the European Economic Area (EEA) and United Kingdom collected by EudraVigilance in the years 2019–2024. The total number of cases signaled for adults (18–64 years) and elders (65–85 years) is 260.

Adverse Reaction	Number and % of Serious ICSRs in the Age Group of 18–64 Years (N = 210)	Number and % of Serious ICSRs in the Age Group of 65–85 Years (N = 50)	Significance Level (P)
Increased blood pressure	26 (12.4%)	17 (34.0%)	0.000490 *
Dissociation/ dissociative disorder	37 (17.6%)	5 (10.0%)	0.270536
Suicidal ideation	23 (10.9%)	3 (6.0%)	0.431401
Anxiety	20 (9.5%)	3 (6.0%)	0.608985
Dizziness	12 (5.7%)	6 (12.0%)	0.206359
Completed suicide	16 (7.6%)	1 (2.0%)	0.260073
Drug ineffective	11 (5.2%)	6 (12.0%)	0.155604
Suicide attempt	12 (5.7%)	2 (4.0%)	0.893348
Loss of consciousness	10 (4.8%)	3 (6.0%)	1.0
Hallucination	11 (5.2%)	0 (0.0%)	N.A.
Generalized tonic–clonic seizure	5 (2.4%)	2 (4.0%)	0.881104
Diplopia	6 (2.8%)	1 (2.0%)	0.881104
Aggression	4 (1.9%)	1 (2.0%)	0.596922
Bradycardia	4 (1.9%)	1 (2.0%)	0.596922

Only adverse reactions of people aged 18–64 years or 65–85 years and signaled more than 4 times are included in the table. N.A. = not applicable. * = *p* < 0.05 vs. Age group of 18–64 years.

**Table 3:** Cases of death not including complete suicide as serious suspected adverse reactions (SARs) in individual case safety reports (ICSRs) associated with esketamine use in the European Economic Area (EEA) and the United Kingdom displayed in EudraVigilance for the years 2019–2024 according to age and sex distribution.

	Cases and % of ICSRs (0–85 Years) (N = 265)	Cases and % of ICSRs (18–64 Years) (N = 210)	Cases and % of ICSRs (65–85 Years) (N = 50)	Male Cases and % of Total ICSRs (N = 265)	Female Cases and % of Total ICSRs (N = 265)
Cases of death	10 (3.8%)	8 (3.8%)	1 (2.0%)	6 (2.3%)	4 (1.5%)

**Table 4:** Reporting odds ratio (ROR) of serious suspected adverse reactions (SARs) to esketamine signaled in the Individual Cases Safety Reports from the European Economic Area and the United Kingdom in the years 2019–2024, aggregated according to the System Organ Class (SOC) levels and compared to SARs related to fluoxetine and venlafaxine in the same years.

SOC	SARs to Esketamine	All Other SARs to Esketamine	SARs to Fluoxetine	All Other SARs to Fluoxetine	ROR Esketamine vs. Fluoxetine (95% C.I.)	SARs to Venlafaxine	All Other SARs to Venlafaxine	ROR Esketamine vs. Venlafaxine (95% C.I.)
Psychiatric disorders	117	148	318	919	2.28 (1.74–3.00)	573	1758	2.42 (1.87–3.15)
Nervous system disorders	26	239	371	695	0.20 (0.13–0.31)	793	1226	0.17 (0.11–0.25)
Vascular disorders	23	242	66	1000	1.44 (0.88–2.36)	156	1863	1.13 (0.72–1.79)
Investigations	18	247	119	947	0.58 (0.35–0.97)	246	1773	0.52 (0.32–0.86)
Respiratory, thoracic and mediastinal disorders	14	251	76	990	0.73 (0.40–1.31)	197	1822	0.51 (0.30–0.90)
General disorders and administration site conditions	14	251	240	826	0.19 (0.11–0.33)	492	1527	0.17 (0.10–0.30)
Cardiac disorders	9	256	96	970	0.35 (0.18–0.71)	258	1761	0.24 (0.12–0.47)
Immune system disorders	9	256	15	1051	2.46 (1.06–5.69)	11	2008	6.42 (2.63–15.63)
Gastrointestinal disorders	7	258	147	919	0.18 (0.08–0.37)	261	1758	0.18 (0.08–0.39)

In brackets are 95% confidence intervals (C.I.’s).

**Table 5:** Reporting odds ratio (ROR) of serious adverse events of suicidal ideation, suicide attempts and completed suicide suspected to be caused by esketamine, fluoxetine, and venlafaxine, signaled in the Individual Cases Safety Reports from the European Economic Area and the United Kingdom in the years 2019–2024.

Adverse Reaction	Esketamine Cases/Not Cases Total Number of Cases = 265	Fluoxetine Cases/Not Cases Total Number of Cases = 1066	Venlafaxine Cases/Not Cases Total Number of Cases = 2019	ROR Esketamine vs. Fluoxetine (95% C.I.)	ROR Esketamine vs. Venlafaxine (95% C.I.)	ROR Fluoxetine vs. Venlafaxine (95% C.I.)
Suicidal ideation	26/239	38/1028	41/1978	2.94 (1.75–4.94)	5.25 (3.15–8.73)	1.78 (1.14–2.79)
Suicide attempt	14/251	70/996	102/1917	0.79 (0.44–1.43)	1.05 (0.59–1.86)	1.32 (0.96–1.81)
Completed suicide	17/248	9/1057	13/2006	8.05 (3.55–18.3)	10.58 (5.08–22.04)	1.31 (0.56–3.08)

In brackets are 95% confidence intervals (C.I.’s).

## Data Availability

The data analyzed and presented in this study are available on the public EudraVigilance data system.
